# Identification of key residues involved in the neuraminidase antigenic variation of H9N2 influenza virus

**DOI:** 10.1080/22221751.2021.1879602

**Published:** 2021-02-02

**Authors:** Fei Wang, Jinsen Wu, Yajuan Wang, Zhimin Wan, Hongxia Shao, Kun Qian, Jianqiang Ye, Aijian Qin

**Affiliations:** a Ministry of Education Key Lab for Avian Preventive Medicine, Yangzhou University, Jiangsu, People’s Republic of China; b Jiangsu Co-innovation Center for Prevention and Control of Important Animal Infectious Diseases and Zoonoses, Jiangsu, People’s Republic of China; c Joint International Research Laboratory of Agriculture and Agri-Product Safety of Ministry of Education of China, Yangzhou University, Jiangsu, People’s Republic of China

**Keywords:** Influenza virus, H9N2, neuraminidase, monoclonal antibodies, key residues, antigenic change

## Abstract

Influenza A H9N2 virus causes economic loss to the poultry industry and has likely contributed to the genesis of H5N1 and H7N9 viruses. The neuraminidase (NA) of H9N2 virus, like haemagglutinin, is under antibody selective pressure and may undergo antigenic change; however, its antigenic structure remains to be elucidated. In this study, we used monoclonal antibodies (mAbs) to probe the H9N2 viral NA residues that are key for antibody binding/inhibition. These mAbs fell into three groups based on their binding/inhibition of the NA of H9N2 viruses isolated during 1999–2019: group I only bounded the NA of the early 2000 H9N2 viruses but possessed no neutralizing ability, group II bounded and inhibited the NA of H9N2 viruses isolated before 2012, and group III reacted with most or all tested H9N2 viruses. We showed that NA residue 356 is key for the recognition by group I mAbs, residues 344, 368, 369, and 400 are key for the binding/inhibition of NA by group II antibodies, whereas residues 248, 253, and the 125/296 combination are key for neutralizing antibodies in group III. Our findings highlighted NA antigenic change of the circulating H9N2 viruses, and provided data for a more complete picture of the antigenic structure of H9N2 viral NA.

## Introduction

H9N2 avian influenza virus (AIV) has been prevalent in domestic poultry in China since its first outbreak in 1992 [1]. When co-infecting with other pathogens, field strains of H9N2 AIV may result in high mortality in poultry [2,3]. There is no evidence for sustainable transmission of H9N2 virus among humans [4], but sporadic human infections have been reported in multiple countries [5–7]. H9N2 AIV has also acted as a donor of the viral internal genes for the genesis of the highly pathogenic H5N1 as well as the novel H7N9 and H10N8 viruses [8–11], which are of significance to the public health. Moreover, H9N2 AIV can also acquire gene segments from H7N9 and H10N8 influenza virus [12].

Haemagglutinin (HA) and neuraminidase (NA) are the most abundant surface proteins and elicit protective immunity against AIV. Both proteins are evolving to evade the host immunity, especially the antibody selective pressure. While there have been numerous research studies on antigenic changes in the HA of H9N2 viruses [13–17], investigations on H9N2 viral NA are relatively sparse [18,19]. Previous studies on H3N2 viruses have shown that mutations in NA may lead to antigenic drift and help virus escape from protective antibodies [20–22]. A detailed antigenic mapping, especially of the NA, may also help monitor the evolution of H9N2 AIV and facilitate the implementation of more effective control strategies.

We have previously defined three NA amino acids that have profound impact on the binding of H9N2 viral NA by two mouse monoclonal antibodies (mAbs) [18]. In the present study, we generated a larger panel of 22 mAbs against the NA of H9N2 AIV and used these antibodies to map the NA residues that are key for antibody binding of H9N2 viral NA and inhibition of the NA enzymatic activity. Our findings from this more detailed NA antigenic mapping may facilitate the NA antigenic characterization and the control of H9N2 AIVs.

## Materials and methods

### Cells, viruses and plasmid

Mouse myeloma SP2/0 cells and hybridoma cells were maintained in Dulbecco’s modified Eagle medium (DMEM, Gibco) supplemented with 15% foetal bovine serum (FBS, Gibco), hypoxanthine and thymidine (HT, Sigma-Aldrich) at 37°C in 5% CO_2_. Madin-Darby canine kidney (MDCK) cells and COS-1 cells were maintained in DMEM supplemented with 10% FBS at 37°C in 5% CO_2_. All field strains of H9N2 viruses were isolated from poultry in China and grown in 9-day-old embryonated SPF chicken eggs. Allantoic fluid of each virus was harvested at 120 h post-inoculation and stored at −70°C. The pCAGGS plasmid, containing NA gene sequence of a wild-type (WT) A/Chicken/Jiangsu/XXM/1999 (XXM) H9N2 virus, was constructed, as previously reported [23].

### Mabs preparation and purification

mAbs in this study were prepared, as previously reported [18]. Splenocytes, from mice immunized with the XXM H9N2 virus, were fused with SP2/0 cells. Hybridomas were screened with COS-1 cells transfected with pCAGGS-NA plasmid in immunofluorescence assay (IFA). The isotype of each mAb was determined with rapid ELISA mouse mAb isotyping kit (Thermo Scientific). Ascitic fluid of each mAb was prepared in 8-week-old mice and purified with protein G column (GE healthcare). For mice experiments, 6-week-old and 8-week-old female BALB/c mice were purchased from Experimental Animal Center of Yangzhou University (Yangzhou, China). All animal experiments were done in accordance with the institutional animal care guidelines, and the protocol (number 06R015) was approved by the Animal Care Committee at Yangzhou University.

### Microneutralization (MN) assay

Neutralizing ability of mAbs was measured in MN assay. Ascitic fluid of each mAb was diluted with an opti-MEM medium that contained 2 μg/mL TPCK-treated trypsin (Sigma–Aldrich), and mixed with 100 median infectious doses (TCID_50_) of XXM H9N2 virus. The mixtures were incubated at 37°C for 30 min and then added into MDCK cells in 96-well plates. Three days later, the supernatants were collected and tested in HA assay. The reciprocal of antibody dilution that resulted in negative HA reading was defined as the MN titre.

### NA gene sequencing and alignment

Viral RNA of H9N2 AIVs was extracted from allantoic fluid with RNA mini kit (Corning). Reverse transcription and PCR for NA gene amplification was carried out, as described in [18]. PCR products were sequenced by BGI, Shanghai, China. Nucleotide and amino acid sequence data were analysed with Lasergene software (www.dnastar.com).

The phylogenetic tree was generated by MEGA X (www.megasoftware.net) with NA amino acid sequences of 19 field strains (accession numbers: ATY41019.1, QJS41644.1 and QJT93521.1 to QJT93537.1), 3 commercial vaccine strains (accession numbers: AAY52598.1, AAP04509.1 and ABH05674.1) and 7 reference strains (accession numbers: AAD49004.1, AAD49006.1, AAD49001.1, AAD49008.1, AKQ43965.1, AKA60605.1 and AGK23860.2).

A total of 2255 NA sequences from H9N2 field strains isolated in China (as of 16 June 2020) were downloaded from GenBank and analysed with multiple sequence alignments by MEGA X (www.megasoftware.net). The percentage of each amino acid, at positions where mutations occurred, was calculated with Excel (Microsoft).

### Site-directed mutagenesis

The 2 ng of plasmid pCAGGS-NA (N356), which expresses WT NA of XXM H9N2 virus, was used as template to construct the plasmid pCAGGS-NA (D356) with super-fidelity DNA polymerase (Vazyme). A pair of primers, N356D-F (GTGGGCCTTTGACGATGGAAATGATATTTGGATG) and N356D-R (CAAATATCATTTCCATCGTCAAAGGCCCACCCTT), was used to introduce N356D mutation into the plasmid pCAGGS-NA (N356). The parameters of the PCR were described as follows: one cycle of 95°C for 3 min, followed by fifteen cycles of 95°C for 15 s, 63°C for 15 s and 72°C for 3 min, and then one cycle of 72°C for 10 min. The PCR product was mixed with Dpn I enzyme (Thermo Scientific) after gel extraction to eliminate template DNA, and directly transformed into competent DH5a cells (Vazyme). Mutant plasmids were sequenced by BGI, Shanghai, China.

### Selection of mAb escape mutants

The selection of mAb escape mutants was done, as previously described [18]. The 50 μL allantoic fluid of XXM H9N2 virus was incubated with 0.5 mL mAb at 37°C for 30 min and inoculated into five 9-day-old SPF embryonated eggs. The allantoic fluid was detected with HA assay and NA sequencing at 120 h post-inoculation.

To obtain monoclonal mutant viruses, the plaque assay was used for viral purification. Viruses with mutant sites were diluted in 10 million folds with Opti-MEM containing 2μg/mL TPCK-treated trypsin and incubated with MDCK cells in a 6-well plate for 60 min. After washing three times with PBS, the infected cells were overlaid with Opti-MEM (Gibco) containing 1% agarose and 2μg/mL TPCK-treated trypsin. The agaroses were coloured with 1% neutral red at 72 h post-infection. Single plaques were selected for expanding culture in fresh MDCK cells. Monoclonal mutant viruses were verified with NA sequencing to make sure no heterozygous peaks at mutant positions in sequencing reports.

### Immunofluorescence assay (IFA)

To determine the specificity of each mAb to field strains and escape mutants, MDCK cells were infected with virus and fixed at 48 h post-infection with cold acetone-alcohol (3:2, vol/vol). Each mAb was diluted and incubated with fixed cells for 30 min incubation at 37°C. Cells were washed 3 times with PBS and incubated with goat anti-mouse-IgG(H + L) antibody conjugated with FITC (Jackson Immunoresearch) for 30 min. After washing with PBS for 3 times, cells were observed under fluorescence microscopy (Olympus) and representative images were taken.

To determine the reactivity of mAbs to mutant NAs, COS-1 cells were transfected with mutant plasmids and fixed in the same way at 48 h post-transfection, and examined in IFA with each mAb and alexa flour 594 conjugated goat anti-mouse-IgG (Fcγ fragment) antibody (Jackson Immunoresearch).

### Neuraminidase inhibition (NI) assay

The NI activity of each mAb to WT XXM H9N2 virus and escape mutants was measured by enzyme-linked lectin assay (ELLA), as previously described [18]. Fetuin (Sigma-Aldrich) was coated onto 96-well plates overnight. mAbs were serially diluted and mixed with a pre-determined amount of virus. The mAb-virus mixtures were added to fetuin-coated wells and incubated at 37°C for 16–18 h. After washing with PBST for 6 times, peroxidase-conjugated peanut agglutinin (PNA-HRP) (Sigma-Aldrich) was added and incubated at room temperature for 2 h. Plates were washed with PBST for another 6 times and followed by the addition of tetramethylbenzidine (TMB) substrate. The reaction was stopped with 1% SDS and absorbance at OD_650_ was read. All statistical analyses were performed with Graphpad Prism version 5 (www.graphpad.com).

## Results

### Generation and characterization of H9N2 viral NA-specific mAbs

Twenty-two hybridomas, which secreted H9N2 viral NA-specific mAbs, were generated from the fusion of mouse myeloma SP2/0 cells with splenocytes from mice immunized with XXM H9N2 virus. The NA sequence of XXM H9N2 virus is highly homologous to the currently used commercial vaccine strain A/Chicken/Shanghai/F/1998 (H9N2). These mAbs all belong to the IgG isotype. The NA-specificity was confirmed by the binding to the NA of XXM H9N2 virus transiently expressed on COS-1 cells in IFA. Six of the 22 mAbs were proved to have neutralizing ability to XXM H9N2 virus in MN assay (Table 1).

**Table 1.** Biological properties of H9N2 viral NA-specific mAbs generated in this study.

mAb^a^	Isotype	IFA titre	Neutralizing titre^b^
A1C4	IgG_1_	51,200	-
A1F1	IgG_2b_	12,800	-
A1G1	IgG_2a_	51,200	-
A2A3	IgG_1_	3200	200
A3C9	IgG_1_	25,600	100
A3H3	IgG_2a_	6400	-
A4C6	IgG_2b_	51,200	800
A5C9	IgG_1_	3200	-
A5D12	IgG_2b_	51,200	400
A6A7	IgG_1_	51,200	100
A6D1	IgG_2a_	6400	-
A6D6	IgG_1_	6400	-
A7E6	IgG_3_	800	-
A7G7	IgG_1_	12,800	-
A7G8	IgG_1_	51,200	-
B1A11	IgG_1_	51,200	-
B2B3	IgG_1_	51,200	-
B2B6	IgG_1_	51,200	-
B2G2	IgG_2a_	3200	-
B4D6	IgG_1_	6400	100
B5B3	IgG_1_	1600	-
B6G5	IgG_1_	51,200	-

^a^ Untreated mouse ascitic ﬂuid of each hybridoma was used in IFA and MN assays.

^b^ The mAbs with no neutralization at the minimum dilution (100-fold dilution) were marked with “-”.

**Figure 1.**
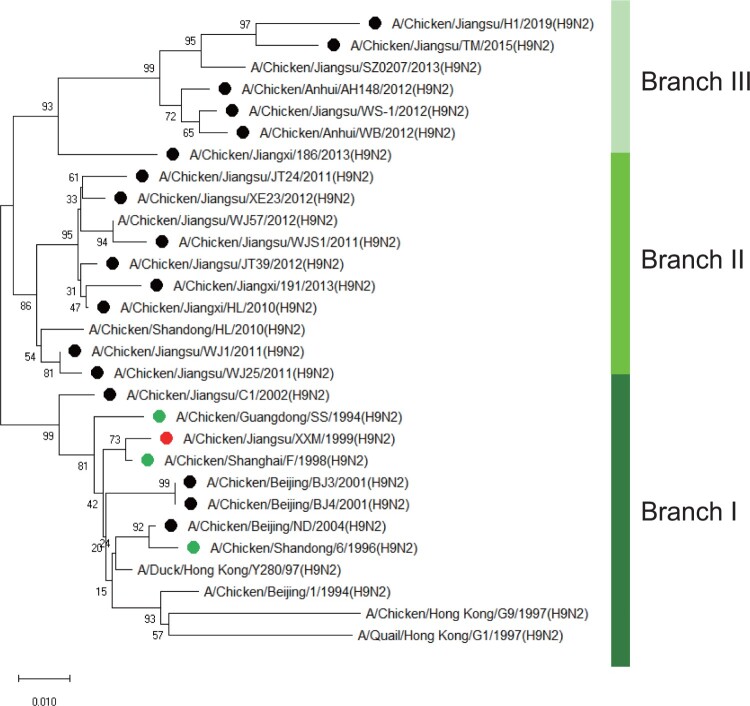
Phylogenetic analysis of NA genes of 19 H9N2 field strains isolated from 1999 to 2019. Green dots represent vaccine strains. Red dot represents XXM H9N2 virus and black dots represent the other 18 field strains used in this study. The phylogenetic tree was constructed with MEGA X in neighbour-joining method and 1000 boot-strap replicates.

To further characterize these mAbs, 19 H9N2 field strains, isolated in China from 1999 to 2019, were used in the subsequent assays. NA gene of field strains formed three branches in the alignment analysis with MEGA X (Figure 1). The first branch consisted of vaccine-like H9N2 viruses and the second branch was mainly made of by H9N2 viruses isolated around 2010s. The third branch was composed of H9N2 viruses isolated since 2012. The reactivity of the 22 antibodies to all field strains was examined in IFA. As shown in Table 2, 4 mAbs, i.e*.* A1F1, A7E6, B2B3 and B5B3, reacted only with the viruses in branch I, whereas another 4 antibodies, A2A3, A4C6, A5D12 and B4D6, reacted with viruses in branch I and branch II but not viruses in branch III. The remaining antibodies, such as A3C9 and A6A7, reacted with almost all 19 H9N2 field strains. Based on the binding patterns, these antibodies were referred to as groups I, II and III, respectively. As measured in MN assay, group II mAbs all possessed neutralizing ability, while group I viruses did not (Table 1). Interestingly, the binding spectrum of mAbs in these groups (Table 2) was consistent with the branching of the NA sequences in the phylogenetic tree (Figure 1).

**Table 2.** Reactivity of 22 N2-specific mAbs to 19 field strains of H9N2 isolated from 1999 to 2019.

Field strain		Group I mAbs				Group II mAbs				Group III mAbs													
		A1F1	A7E6	B2B3	B5B3	A2A3	A4C6	A5D12	B4D6	A1C4	A1G1	A3C9	A3H3	A5C9	A6A7	A6D1	A6D6	A7G7	A7G8	B1A11	B2B6	B2G2	B6G5
Branch I	A/Chicken/Beijing/ND/2004(H9N2)	**+**	**+**	**+**	**+**	**+**	**+**	**+**	**+**	**+**	**+**	**+**	**+**	**+**	**+**	**+**	**+**	**+**	**+**	**+**	**+**	**+**	**+**
	A/Chicken/Beijing/BJ4/2001(H9N2)	**+**	**+**	**+**	**+**	**+**	**+**	**+**	**+**	**+**	**+**	**+**	**+**	**+**	**+**	**+**	**+**	**+**	**+**	**+**	**+**	**+**	**-**
	A/Chicken/Beijing/BJ3/2001(H9N2)	**+**	**+**	**+**	**+**	**+**	**+**	**+**	**+**	**+**	**+**	**+**	**+**	**+**	**+**	**+**	**+**	**+**	**+**	**+**	**+**	**+**	**-**
	A/Chicken/Jiangsu/XXM/1999(H9N2)	**+**	**+**	**+**	**+**	**+**	**+**	**+**	**+**	**+**	**+**	**+**	**+**	**+**	**+**	**+**	**+**	**+**	**+**	**+**	**+**	**+**	**+**
	A/Chicken/Jiangsu/C1/2002(H9N2)	**-**	**-**	**-**	**+**	**+**	**+**	**+**	**+**	**-**	**+**	**+**	**+**	**+**	**+**	**+**	**+**	**+**	**+**	**+**	**+**	**+**	**+**
Branch II	A/Chicken/Jiangsu/WJ25/2011(H9N2)	**-**	**-**	**-**	**-**	**+**	**+**	**+**	**+**	**+**	**+**	**+**	**+**	**+**	**+**	**+**	**+**	**+**	**+**	**+**	**+**	**+**	**+**
	A/Chicken/Jiangsu/WJ1/2011(H9N2)	**-**	**-**	**-**	**-**	**+**	**+**	**+**	**+**	**+**	**+**	**+**	**+**	**+**	**+**	**+**	**+**	**+**	**+**	**+**	**+**	**+**	**+**
	A/Chicken/Jiangxi/HL/2010(H9N2)	**-**	**-**	**-**	**-**	**+**	**+**	**+**	**+**	**-**	**+**	**+**	**+**	**+**	**+**	**-**	**+**	**+**	**+**	**+**	**+**	**+**	**-**
	A/Chicken/Jiangxi/191/2013(H9N2)	**-**	**-**	**-**	**-**	**+**	**+**	**+**	**+**	**-**	**+**	**+**	**+**	**+**	**+**	**-**	**-**	**+**	**+**	**+**	**-**	**+**	**+**
	A/Chicken/Jiangsu/WJS1/2011(H9N2)	**-**	**-**	**-**	**-**	**+**	**+**	**+**	**+**	**+**	**+**	**+**	**+**	**+**	**+**	**+**	**-**	**+**	**+**	**+**	**+**	**+**	**+**
	A/Chicken/Jiangsu/XE23/2012(H9N2)	**-**	**-**	**-**	**-**	**+**	**+**	**+**	**+**	**+**	**+**	**+**	**+**	**+**	**+**	**+**	**+**	**+**	**+**	**+**	**+**	**+**	**+**
	A/Chicken/Jiangsu/JT39/2012(H9N2)	**-**	**-**	**-**	**-**	**+**	**+**	**+**	**+**	**+**	**+**	**+**	**+**	**+**	**+**	**+**	**+**	**+**	**+**	**+**	**+**	**+**	**+**
	A/Chicken/Jiangsu/JT24/2011(H9N2)	**-**	**-**	**-**	**-**	**+**	**-**	**-**	**-**	**+**	**+**	**+**	**+**	**+**	**+**	**+**	**+**	**+**	**+**	**+**	**+**	**+**	**+**
Branch III	A/Chicken/Jiangxi/186/2013(H9N2)	**-**	**-**	**-**	**-**	**-**	**-**	**-**	**-**	**+**	**+**	**+**	**+**	**+**	**+**	**+**	**+**	**+**	**+**	**+**	**+**	**+**	**+**
	A/Chicken/Anhui/WB/2014(H9N2)	**-**	**-**	**-**	**-**	**-**	**-**	**-**	**-**	**+**	**+**	**+**	**+**	**+**	**+**	**+**	**+**	**+**	**+**	**+**	**+**	**+**	**+**
	A/Chicken/Jiangsu/WS-1/2012(H9N2)	**-**	**-**	**-**	**-**	**-**	**-**	**-**	**-**	**+**	**+**	**+**	**+**	**+**	**+**	**+**	**+**	**+**	**+**	**+**	**+**	**+**	**-**
	A/Chicken/Anhui/AH148/2012(H9N2)	**-**	**-**	**-**	**-**	**-**	**-**	**-**	**-**	**+**	**+**	**+**	**+**	**+**	**+**	**+**	**+**	**+**	**+**	**+**	**+**	**+**	**-**
	A/Chicken/Jiangsu/TM/2015(H9N2)	**-**	**-**	**-**	**-**	**-**	**-**	**-**	**-**	**+**	**+**	**+**	**+**	**+**	**+**	**+**	**+**	**+**	**+**	**+**	**+**	**+**	**-**
	A/Chicken/Jiangsu/H1/2019(H9N2)	**-**	**-**	**-**	**-**	**-**	**-**	**-**	**-**	**+**	**+**	**+**	**+**	**+**	**+**	**+**	**+**	**+**	**+**	**+**	**+**	**+**	**+**

All viruses were listed in the NA phylogenetic branching order.

Positive results of IFA were labelled as + and negative ones were labelled as -.

### A single mutation N356D impacted the binding of group I mAbs to NA

**Figure 2.**
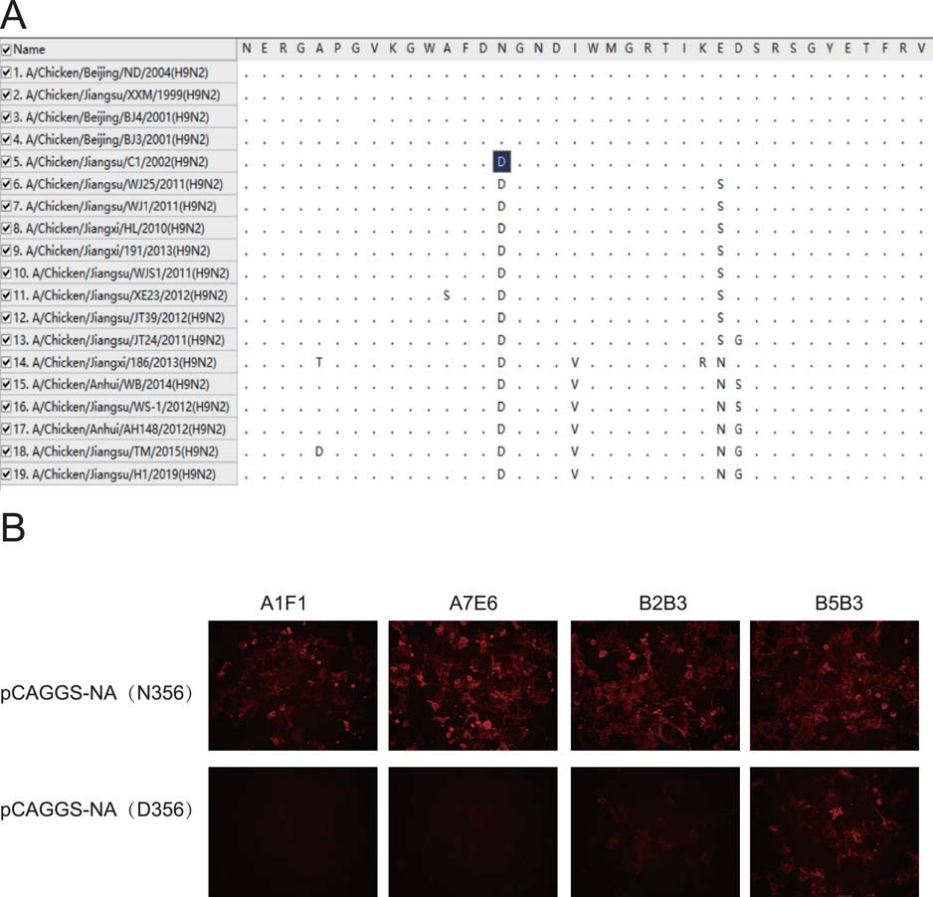
The impact of N356D mutation on reactivity of group I mAbs. (A) Amino acid sequence alignment of NA sequences (342–379, N2 numbering) of 19 field strains was analysed with MEGA X. The D356 in NA of A/Chicken/Jiangsu/C1/2002 (H9N2) virus was marked in dark blue. (B) Plasmids of WT NA gene (pCAGGS-NA (N356)) and mutant NA gene (pCAGGS-NA (D356)) were transfected into COS-1 cells and examined with 4 mAbs in IFA.

Group I mAbs A1F1, A7E6 and B2B3 only reacted with the viruses belonging to branch I in IFA except the A/Chicken/Jiangsu/C1/2002 H9N2 virus. Moreover, all group I mAbs failed to bind the NA of the viruses in branches II and III. Sequence alignment revealed that the only difference between the NA of these viruses is that of A/Chicken/Jiangsu/C1/2002 bears D356 in the NA, while the other isolates in branch I possess N356 (Figure 2(A)).

To examine whether the N356D mutation was accounted for the loss of binding of NA by group I mAbs, pCAGGS plasmids, expressing the WT XXM H9N2 viral NA (N356) or mutant NA that carries D356, were constructed, and COS-1 cells were transfected with these plasmids to transiently express the WT or mutant NA on cells. In IFA, COS-1 cells transfected with pCAGGS-NA (D356) were not recognized by mAbs A1F1 or A7E6, and were only weakly bound by mAb B2B3 (Figure 2(B)). These data demonstrate that residue 356 was the key contact in the epitope(s) targeted by group I mAbs. Residue 356 locates at the back of NA head (Figure 5(B)), which is unlikely in a neutralizing epitope. Consistent with this concept, group I mAbs did not neutralize virus in MN assay (Table 1). Among the ∼2200 Chinese H9N2 viral NA sequences available in Genbank, ∼63% possessed N356D mutation (Table 3), implying that the epitope(s) targeted by group I mAbs has been under the antibody selective pressure in the field.

**Table 3.** Natural substitutions at 9 key amino acid positions of H9N2 AIV field strains isolated in China.

Amino acid position	Substitutions (%)^a^
125	S(61.73), G(34.9), D(2.88), I^b^,N
248	G(99.37), E, R
253	R(98.18), K(1.64), G
296	K(90.16), R(9.49), N, M
344	R(98.18), S(1.15), E, K
356	D(63.19), S(19.07), N(14.27), Y(1.59), V(1.46)
368	N(41.24), K(27.54), S(17.74), E(8.34), D(3.33), V(1.20), R
369	D(61.60), G(32.54), N(4.66), K, C, E
400	S(90.55), R(4.48), N(3.56), G, I, H, T

^a^ A total of 2255 NA sequences of Chinese H9N2 isolates available in GenBank (as of 16 June, 2020) were analysed.

^b^ Proportions of amino acids less than 1% were not shown.

### Multiple residues identified in the NA of escape mutants selected by group II mAbs

Group II mAbs A2A3, A4C6, A5D12 and B4D6 bounded the NA of branches I and II viruses in IFA and have the neutralizing ability in MN assay. To identify the key residues in the epitope(s) recognized by these mAbs, escape mutants of XXM H9N2 virus were selected with each antibody (Table S1). Mutations at residues 344, 368, 369 and 400 were identified in the resultant escape mutants. Interestingly, mutations at amino acid position 369 in the NA were present in mutant viruses selected by all group II mAbs. Mutations E368 K, R344I and R344 K were also found in the mutants selected with mAb A2A3. In addition, another 2 mutants, selected by mAbs A4C6 and A5D12, both bear an S400R mutation in the NA. The 6 different escape mutants of selected viruses were purified in plaque assay for further assays. IFA, using MDCK cells that were infected with the WT XXM H9N2 virus and each escape mutant, confirmed the impact of each amino acid mutations on NA binding by the mAbs (Figure S1). The IFA results demonstrate that not all selected mutants lost the binding by mAbs, implying that the footprints of the 4 mAbs may overlap, but they do not recognize the same epitope.

**Figure 3.**
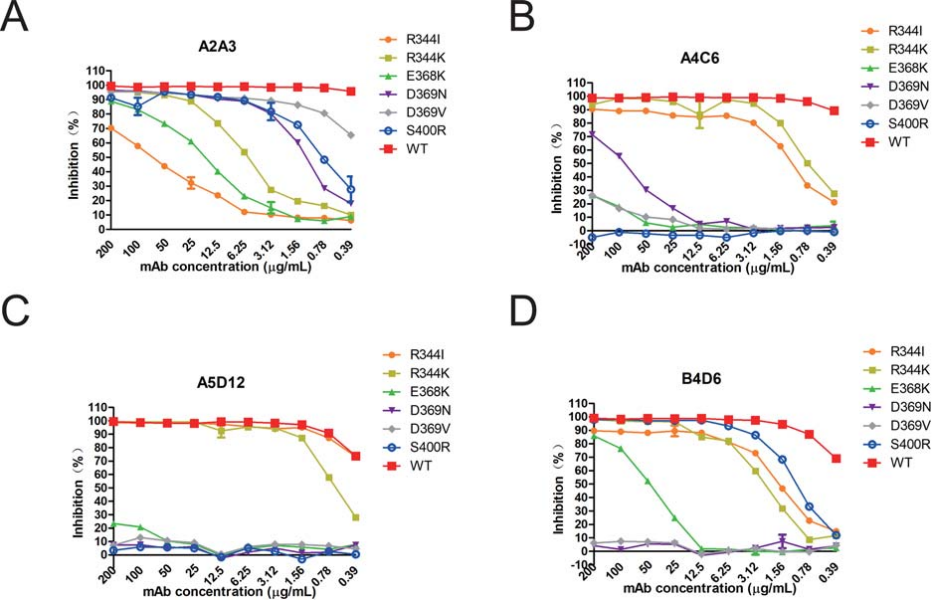
NI ability of group II mAbs to escape mutants and WT XXM H9N2 virus. NA inhibitory effect of mAbs A2A3, A4C6, A5D12 and B4D6 on escape mutants was measured in ELLA. A to D represent results of A2A3, A4C6, A5D12 and B4D6.

The impact of the identified amino acid mutations, on inhibition of the NA activity by the group II mAbs, was examined with ELLA (Figure 3(A–D)). Interestingly, mutations at residue 344 had more profound impact on the inhibition by mAb A2A3 than by other mAbs, while mutation S400R resulted in more complete loss of inhibition by mAbs A4C6 and A5D12 than by A2A3 and B4D6. Furthermore, mutations at residues 368 and 369 impacted the inhibition by all group II mAbs, with reduction in inhibition by antibodies A4C6, A5D12 and B4D6 being more significantly.

All mutant positions, selected by group II mAbs, locate around the NA active centre (Figure 5(A)), which make up overlapping epitopes. Residues R344 and S400, both account for over 90%, are relatively conserved in all H9N2 NA sequences of Chinese isolates (Table 3). However, amino acids at positions 368 and 369 are of variety. Especially the substitution of N368 introduces a potential *N*-glycosylation site at the periphery of NA active centre.

### Key NA residues for the binding/inhibition of NA by group III mAbs

The majority of our mAbs, i.e*.* those in group III, exhibited broad reactivity to most or all of the H9N2 viruses tested. Among these, mAbs A3C9 and A6A7 possess neutralizing ability to XXM H9N2 virus in MN assay (Table 1). Escape mutants of XXM H9N2 virus were also selected to determine key residues in the epitopes recognized by these two mAbs, Mutations G125D/K296N, K296N and R253 K were identified in mutants selected by mAb A3C9, a mutant with the single mutation G248E in NA was selected with mAb A6A7 (Table S2).

**Figure 4.**
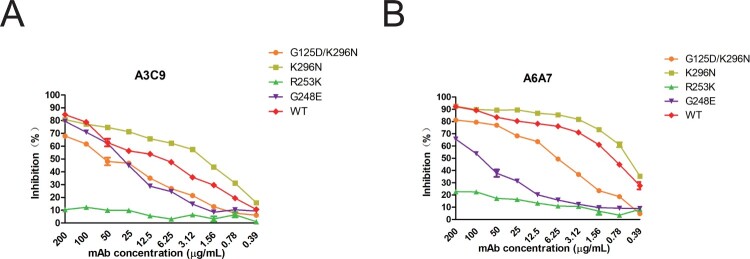
NI ability of group III mAbs A3C9 and A6A7 on escape mutants and WT XXM H9N2 virus. NA inhibitory effect of mAbs A3C9 and A6A7 on escape mutants was measured in ELLA. A represents result of A3C9 and B represents result of A6A7.

In IFA, mAb A3C9 exhibited reduced binding to mutant viruses, containing G125D/K296N or R253 K mutations in the NA (Figure S2). mAb A6A7 did not bind the mutant that bears the R253 K mutation. NI effect of mAbs A3C9 and A6A7 on the four mutants was also measured by ELLA (Figure 4(A and B)). Interestingly, mAbs A3C9 and A6A7 inhibited mutant NA with the K296N mutation slightly more efficiently than the WT NA, while both antibodies have reduced inhibition on mutant NA with the double-mutation G125D/K296N. Furthermore, the inhibition on NA with G248E or R253 K was significantly lower than on the WT NA. These results indicated that the epitopes bound by mAbs A39 and A6A7 are overlapping to some extent.

**Figure 5.**
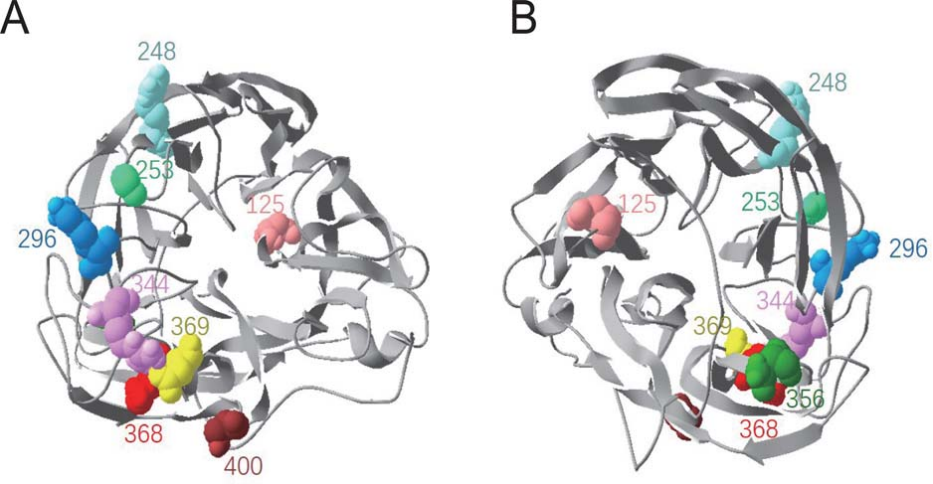
Locations of the 9 identified key residues on NA. The NA head structure of N2 subtype (PDB:1NN2) was analysed with Swiss PDB Deep-viewer. The 9 residues were labelled with various colours in an NA monomer. The structure is shown in the top view (A) and bottom view (B).

Residues 125, 248, 253 and 296, which are distributed at the bottom of or in close proximity to the NA active centre (Figure 5(A and B)), make up a new overlapping region which is distinct from the region identified by group II mAbs. There have been greater variation at NA positions 125 and 296 (Table 3). Residues at positions 248 and 253, which exhibited a significant impact on antibody binding and inhibition of NA, have been very conserved, with G248 and R253 present in >98% of the analysed H9N2 viral NAs. These residues, especially at positions 248 and 253, are crucial for universal epitope in NA of H9N2 AIVs.

## Discussion

Antigenic mapping with mAbs serves as an important way for characterizing influenza viral HA and NA, as it helps find molecular makers for monitoring the antigenic change of these ever-evolving antigens. It also facilitates the identification of important epitopes or antigenic sites for further detailed X-ray crystal chromatography [24,25]. We have previously identified amino acid positions 198, 199 and 338, which were critical for the binding of H9N2 viral NA by two murine mAbs [18].

In the present study, we used a larger number of mAbs to map the NA residues that are key for antibody binding and inhibition of NA. With these antibodies, which were placed into different groups based on their distinct NA-binding and inhibition properties, 9 key residues were identified, adding data towards a more complete antigenic characterization of H9N2 viral NA.

As expected, the majority of the 9 residues (i.e*.* 125, 248, 253, 296, 344, 356, 368, 369, and 400) are located close to or around the NA active centre, explaining why some of the selecting antibodies were able to inhibit the enzymatic activity of NA. Residues 248, 253, 368, 369 and 400 were also reported to be important for antibody binding of the NA from H2N2, H3N2 and H7N9 viruses [22,24,26–28], and are probably within different antigenic sites or epitopes, i.e*.* residues 248 and 253 are in an antigenic site or epitope, while residues 368, 369 and 400 are in another [28,29].

Residue 296 is in close proximity to the 250-loop that contains residues 248 and 253, and on the H3N2 viral NA this residue is within the epitope recognized by a broadly reactive mAb B10 [22]. Residue 344 is within the 340-loop, which has been reported to be involved in the binding or antigenic drift of NA by some N1, N2, and N9-specific mAbs [20,29,30]. Residue 125 is deep in the NA active site, and when mutated simultaneously with residue 296 (D125G/K296N) had a significant impact on antibody binding/inhibition of NA. Residue 356, identified by group I mAbs in our study, is on the back of the NA head domain. N356 has been reported to be associated with enhanced pathogenicity of H9N2 virus to mice [31]; however, the mechanism, underlying the impact of amino acid mutation at position 356 on antibody binding, remains to be investigated. It is unknown whether residue 356 is in direct contact with antibodies, or amino acid mutation at residue 356 can cause global structural change and thus impacts antibody binding.

Commercial H9N2 vaccines have been used in the poultry in China for over two decades [1]. The circulating H9N2 viruses are, therefore, under continuous selective pressure, which may have resulted in antigenic change in both HA and NA [32]. Among the more than 2200 Chinese H9N2 viral NA sequences analysed in our study, a substantial proportion processed mutations at some of the key residues identified in the present study. For instance, while the H9N2 vaccine strains and earlier isolates carried N356 and E368 in the NA, the majority of the current circulating H9N2 viruses possess D356, N368 or K368. The E368N mutation introduced a potential *N*-linked glycosylation site, which may have a significant impact on the inhibition of NA by serum antibodies. With mutations occurred at multiple key NA residues, which may reduce antibody binding and inhibition activity, NA antigenic change might have occurred in the circulating H9N2 viruses. Such a situation necessitates continuous monitoring of the H9N2 virus evolution and a careful evaluation of its impact on vaccine efficacy in the field.

## Supplementary Material

Figure-6.eps

Figure-3.eps
